# Evaluation of serum levels of Irisin as a marker of endothelial dysfunction in patients with type 2 diabetes mellitus

**DOI:** 10.1002/edm2.403

**Published:** 2023-03-14

**Authors:** Thoraya Mohamed Ahmed, Mahmoud Nassar, Hanaa Ahmed Abdallah Mohamed, Khaled El‐sayed Elhadidy, Hanan Mohamed Farhan, Ahmed Sayed Abd El Basset, Riem M. Elmessiery, Mahmoud Farid Kamel

**Affiliations:** ^1^ Internal Medicine Department at Faculty of Medicine Beni Suef University Beni Suef Egypt; ^2^ Internal Medicine Department at Icahn School of Medicine at Mount Sinai, NYC Health+Hospitals Queens New York USA; ^3^ Clinical and Chemical Pathology Department Faculty of Medicine, Beni Suef University Beni Suef Egypt; ^4^ Radiology Department Faculty of Medicine, Bani‐Suef University Beni Suef Egypt; ^5^ Internal Medicine Department, Kasr Alainy Faculty of Medicine Cairo University Cairo Egypt

**Keywords:** inflammation, insulin resistance, Irisin, obesity, T2DM

## Abstract

**Introduction:**

Insulin resistance and obesity have been associated with irisin, a protein in fat cells. The levels of irisin in patients with type 2 diabetes mellitus (T2DM) were significantly lower than those in non‐diabetics. This study aimed to examine the relationship between serum irisin levels and endothelial dysfunction in patients with T2DM.

**Methods:**

There were 90 participants in this study. We matched 65 patients with T2DM with 25 healthy control participants. A series of tests were performed on the participants, including fasting blood glucose, 2 hours postprandial blood glucose, glycated haemoglobin, triglycerides (TG), total cholesterol, high‐density lipoprotein cholesterol (HDL‐C), low‐density lipoprotein cholesterol (LDL‐C), TG/HDL‐C ratio and albumin/creatinine ratio. In addition to measuring high‐sensitivity C‐reactive protein (hs‐CRP). Enzyme‐linked immunosorbent assay (ELISA) technique was used for estimating irisin concentrations.

**Results:**

Flow‐mediated dilation (FMD) was significantly lower in patients with T2DM; however, there was a non‐statistically significant difference between healthy controls and patients with T2DM regarding serum Irisin level. CRP and LDL levels were inversely correlated with circulating irisin levels. In a stepwise regression analysis, only the hs‐CRP and LDL were statistically significant in predicting irisin level.

**Conclusions:**

In patients with T2DM, serum levels of irisin were inversely correlated with hyperglycaemia, body mass index and per cent body fat; this suggests that detecting irisin levels early can prevent cardiovascular diseases from progressing. According to the study results, serum irisin serves as a predictive marker for early cardiovascular disease, thus preventing the disease from progressing. There is a need for further research in order to understand how irisin contributes to the development of atherosclerosis and the development of diabetic complications.

## Graphical abstract

This study aims to examine the relationship between serum irisin levels and endothelial dysfunction in patients with T2DM. There were 90 participants in this study. We matched 65 patients with T2DM with 25 healthy control participants. In patients with T2DM, serum levels of irisin were significantly correlated with hyperglycemia, BMI, and percent body fat; this suggests that detecting irisin levels early can prevent cardiovascular diseases from progressing.

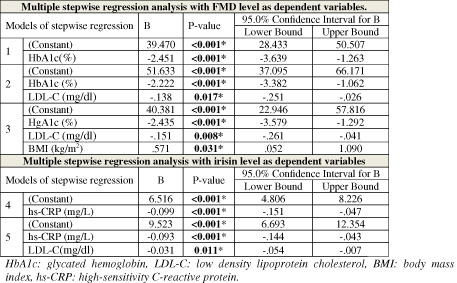

## 1 INTRODUCTION

The prevalence of type 2 diabetes mellitus (T2DM) has been estimated to be 200 million individuals worldwide, and the number is steadily rising.^1^ This issue is one of the world's most pressing public health concerns.^2^ Several risk factors are associated with T2DM, including obesity, dyslipidaemia and insulin resistance.^3^ Diabetes patients are at a 10‐fold greater risk of cardiovascular events than those without diabetes.^2^ Cardiovascular disease is the leading cause of mortality and disability in individuals with T2DM.^4^

Endothelial dysfunction (ED) is one of the first pathological events in the pathogenesis of several vascular disorders, including diabetic vascular complications.^5^ ED is exacerbated by diabetic microvascular and macrovascular consequences.^4^ Prevention and treatment of cardiovascular disease could theoretically benefit from improved endothelial function. Additionally, effective therapy options are limited, and the discovery of new therapeutic targets is highly desirable.^6^

T2DM and its ED complications are often associated with inflammation.^7^ The number of serum indicators for ED in patients with diabetes is limited. These indicators of ED, inflammation and oxidative stress are often investigated. Most of the biomarkers used include serum E‐selectin, endothelial progenitor cells (EPCs), endothelial microparticles (EMPs), tumour necrosis factor‐α/interleukin‐10 (TNF‐α/IL‐10), nitric oxide, malondialdehyde and lipoprotein (a).^8^ Many cytokines influence the inflammatory burden in T2DM, including neuregulin, which has been reported as an early predictor of diabetic complications as microvascular dysfunction is considered the early manifestation of subclinical systemic damage.^9^, ^10^ Omentin, an adipokine found in visceral adipose tissue, is negatively correlated with diabetes, obesity and inflammation.^11^, ^12^ Inflammatory markers such as cardiotrophin, mean platelet volume, serum uric acid, monocyte/lymphocyte ratio and neuregulin have been identified as being associated with inflammation in diabetic chronic complications.^13^, ^14^, ^15^ Urinary kidney injury molecule‐1 levels predict renal injury in diabetic nephropathy at the early stages, regardless of the amount of albuminuria present.^16^ The prevalence of diabetic complications is associated with chronic and low‐grade inflammation and an increase in the ratio of C‐reactive protein to serum albumin.^17^ Irisin is a novel peptide that plays a significant role in human health and disease. Several conditions in which inflammation occurs are alleviated by irisin via its anti‐inflammatory and anti‐apoptotic properties by inhibiting apoptosis and nuclear factor‐B signalling.^18^ Considering that T2DM causes inflammation and that irisin alleviates inflammation, it makes sense to study irisin in individuals with T2DM. In addition to being a myokine and an adipokine secreted by muscles and subcutaneous fat, this hormone is also impacted by physical activity and diet.^19^ Obesity and insulin resistance have been linked to irisin.^20^ Studies have reported that irisin may be a therapeutic molecule for metabolic diseases that can be corrected by exercise. Irisin has been reported to raise total body energy expenditure, promote weight reduction, improve glucose tolerance and relieve insulin resistance.^8^

The reduction of irisin levels has been linked to insulin resistance and the development of T2DM; in addition, irisin may improve insulin sensitivity.^21^ A systematic review was conducted and has indicated that irisin offers new opportunities for understanding and managing obesity, metabolic syndrome and diabetes.^22^ The serum irisin levels of patients with T2DM were substantially reduced compared to the serum irisin levels of non‐diabetic.^23^

The purpose of this case–control study is to determine whether serum irisin levels correlate with ED measured by flow‐mediated dilatation (FMD), body mass index (BMI) and fat per cent in body composition measured by bioelectrical impedance analysis (BIA) among patients with T2DM. In addition, to determine whether irisin levels in serum could be used as a new indicator for ED.

## 2 MATERIALS AND METHODS

### 2.1 Study design and ethics approval

The study was conducted at the Beni‐Suef University Hospital (a tertiary hospital in Egypt), Internal Medicine Department, Diabetes and Endocrine Clinic. The study included 65 patients with T2DM. The subjects were matched with 25 healthy control subjects. The Ethical Review Committee of the Faculty of Medicine, Beni‐Suef University, approved this study. Participants provided written informed consent.

### 2.2 Study population

Individuals with T2DM between the ages of 40 and 60, with varying BMIs, and undergoing either oral treatment (sulfonylurea and/or Dipeptidyl peptidase 4 inhibitors (DPP4I)) or insulin therapy. The study excluded patients with congestive heart failure, valvular heart disease, acute coronary syndrome, atrial fibrillation, smokers, nephrotic syndrome, stroke, acute inflammation, liver disease, kidney disease, thyroid illness, pregnant women and those under 40 years of age. In addition, patients with clinical angiopathy, including micro‐ and macroangiopathy, as well as hypertension, were excluded from the study. Furthermore, patients who received statins, diuretics, immunomodulatory agents, metformin and pioglitazone were excluded from the study.

### 2.3 Data collection and follow‐up

The individuals underwent a full medical history and a clinical examination. All recruited individuals were required to have an office blood pressure (BP) of 130/80 mmHg.

The following anthropometric measurements were taken: weight, height and body mass index (BMI). Bioelectrical Impedance Analysis (BIA). The BIA was conducted using a body fat analyser model No: BT‐905. The participants were advised to avoid eating or drinking within 4 h of the test, to avoid exercising within 12 h h of the test, to urinate within 30 minutes of the test, to avoid drinking alcohol within 48 h of the test, not to take diuretic medications within 7 days prior to the test, and to avoid testing in a premenstrual period.

A routine laboratory investigation was conducted on all participants, including fasting blood glucose (FBG), 2 h postprandial (2hpp) blood glucose, glycated haemoglobin (HbA1c), triglycerides (TG), total cholesterol (TC), high‐density lipoprotein cholesterol (HDL‐C), low‐density lipoprotein cholesterol (LDL‐C) and albumin/creatinine (A/C). Analyses were conducted using the Bechman CX5 automated chemistry analyser. As a surrogate marker for ED, small dense LDL (sdLDL) was calculated through the ratio of TG to HDL‐C. High‐sensitivity C‐reactive protein (hs‐CRP) was quantified using chemiluminescent immunoassay.

### 2.4 Flow‐mediated dilatation measurement

Endothelial function was evaluated by measuring FMD in the right brachial artery with the patient in the supine position and abduction of the right arm. Four experienced technicians conducted evaluations in a dimly lit room for about 30 min. Using B‐mode, the brachial artery diameter was measured continuously for 1 min to obtain the baseline diameter (D1), with the pulsed Doppler signal at a 60 angle to the vessel. Next, a 5 min vascular occlusion period was obtained by inflating a blood pressure cuff on the right forearm up to a pressure of 250 mm Hg. Thereafter, the maximum post‐occlusion vascular diameter (D2) was measured within 3–5 min after deflation of the blood pressure cuff, to avoid the bias of maximum hyperaemia. The FMD value was obtained from the following equation reported by Celermajer et al,^24^: FMD (%) = [(D2‐D1)/D1] × 100, where D1 = baseline diameter and D2 = diameter during reactive hyperaemia.

### 2.5 Estimation of irisin concentration by enzyme‐linked immunosorbent assay (ELISA) technique

Irisin serum concentration was measured using Irisin (Human Fibronectin type III domain‐containing protein 5, Irisin) – ELISA Assay Kit, with a detection range of Irisin: (0.5–7.8 pg/L), according to manufacture instructions.

### 2.6 Statistical analysis

The data were analysed using SPSS v. 22 (Statistical Package for Social Science) for Windows. Quantitative variables were presented as mean + standard deviation (SD), while qualitative variables were presented as numbers (No.) and percentages (%). Quantitative variables were compared using an independent sample t‐test and a one‐way analysis of variance. Categorical data were compared using chi‐square tests. To determine the relationship between the various quantitative variables, a Pearson correlation test was used. A regression analysis was conducted to identify the predictor factors that may affect the level of FMD and irisin. The quantitative data for each of the study groups were tested for normality using a one‐sample Kolmogorov–Smirnov test before being subjected to inferential statistical tests. A *p*‐value was used to evaluate the significance of the results, which can be classified as non‐significant when the *p*‐value is >.05, significant when the *p*‐value is ≤.05, and highly significant when the *p*‐value is ≤.001.

## 3 RESULTS

An overview of the clinical characteristics and biochemical data of diabetic and non‐diabetic subjects can be found in Table 1. Compared to healthy controls, patients with T2DM demonstrated significantly higher levels of hs‐CRP, FBG, 2hpp, Creatinine, LDL‐C, TG, TG/HDL‐C ratio, TC, HbA1c, A/C, BMI and fat percentage than healthy controls. Patients with T2DM demonstrated significantly lower FMD, but serum Irisin levels were not significantly different between healthy controls and patients with T2DM (*p* > .05).

**TABLE 1:** Comparison of Laboratory and subjects' criteria

		Mean	Std. deviation	*p*‐value
Irisin (ng/ml)	Control	3.362	1.3	.978
	Type 2 DM	3.375	1.9	
hs‐CRP (mg/L)	Control	26.681	5.5	.010*
	Type 2 DM	**31.752**	8.2	
FBG (mg/dl)	Control	86.952	11.7	<.001*
	Type 2 DM	**185.67**	43.5	
2‐hpp (mg/dl)	Control	120.14	8.9	<.001*
	Type 2 DM	**264.01**	58.7	
Creatinine (mg/dl)	Control	0.739	0.11	.009*
	Type 2 DM	**0.853**	0.18	
LDL‐C (mg/dl)	Control	79.619	11.4	<.001*
	Type 2 DM	**103.25**	17.4	
TC (mg/dl)	Control	148.91	27	<.001*
	Type 2 DM	**215.9**	44.4	
TG (mg/dl)	Control	116.76	28.4	**.046**
	Type 2 DM	**145.75**	63.5	
HDL‐C (mg/dl)	Control	**81.4**	35.6	<.001*
	Type 2 DM	**62.8**	14.3	
TG/HDL ratio	Control	**1.9**	0.63	.01*
	Type 2 DM	**2.21**	1.6	
FMD (mg/dl)	Control	**27.762**	7.57	<.001*
	Type 2 DM	17.072	9.1	
HBA1c (%)	Control	5.081	0.66	<.001*
	Type 2 DM	**9.139**	1.6	
A/C	Control	18.048	5.1	.025*
	Type 2 DM	**21.776**	6.8	
BMI (kg/m^2^)	Control	22.633	2.4	.001*
	Type 2 DM	**25.536**	3.7	
Fat (%)	Control	25.886	4.7	<.001*
	Type 2 DM	**34.955**	4.3	
Disease duration (years)	Cases (T2DM)	35.76	17.02	
SBP (mmHg)	Cases (T2DM)	124	9	.341
DBP (mmHg)		73	4	

*Note:* Bold denotes significant values.

Abbreviations: 2hPP: Two‐hours post‐prandial blood glucose; A/C: albumin/creatinine; BMI: body mass index; FBG: fasting blood glucose; HbA1c: glycated haemoglobin; HDL‐C: high‐density lipoprotein cholesterol; hs‐CRP: high‐sensitivity C‐reactive protein; LDL‐C: low‐density lipoprotein cholesterol; TC: total cholesterol; TG: triglycerides.

The Pearson correlation analysis, as summarized in (Table 2), showed a statistically significant linear negative correlation between FMD and FBG, 2hpp, LDL‐C, TC, TG, TG/HDL‐C ratio and HbA1c (*p*‐values≤.05). Furthermore, circulating levels of irisin were inversely correlated with levels of hs‐CRP and LDL‐C that were sensitive to high levels of irisin (*p*‐values ≤.05) (Table 3).

**TABLE 2:** : Pearson's correlation analysis between FMD and other studied variables

Independent variables		FMD
Age (years)	*R*	−0.125
	*p*‐value	0.312
Irisin (ng/ml)	*R*	0.154
	*p*‐value	0.212
hs‐CRP (mg/L)	*R*	0.006
	*p*‐value	0.961
FBG (mg/dl)	*R*	**−0.254** ^*^
	*p*‐value	**0.038**
2hpp (mg/dl)	*R*	**−0.248** ^*^
	*p*‐value	**0.043**
Creatinine (mg/dl)	*R*	−0.108
	*p*‐value	0.383
LDL‐C (mg/dl)	*R*	**−0.331** ^**^
	*p*‐value	**0.006**
TC (mg/dl)	*R*	**−0.384** ^**^
	*p*‐value	**0.001**
TG (mg/dl)	*R*	**−0.394** ^**^
	*p*‐value	**0.001**
HDL‐C (mg/dl)	*R*	−0.199
	*p*‐value	0.057
TG/HDL‐C ratio	*R*	**−0.209**
	*p*‐value	0.047*
HbA1c (%)	*R*	**−0.455** ^**^
	*p*‐value	**<0.001**
A/C	*R*	−0.042
	*p*‐value	0.733
BMI (kg/m^2^)	*R*	0.108
	*p*‐value	0.384
Fat (%)	*R*	−0.225
	*p*‐value	0.067
Duration of DM (years)	*R*	0.16
	*p*‐value	0.197

*Note:* Bold denotes significant values.

Abbreviations: 2hPP: Two‐hours post‐prandial blood glucose; A/C: albumin/creatinine; BMI: body mass index; FBG: fasting blood glucose; HbA1c: glycated haemoglobin; HDL‐C: high‐density lipoprotein cholesterol; hs‐CRP: high‐sensitivity C‐reactive protein; LDL‐C: low‐density lipoprotein cholesterol; TC: total cholesterol; TG: triglycerides.

**TABLE 3:** Pearson's correlation analysis between Irisin and other studied variables

Independent variables		Irisin
Age (years)	*R*	0.092
	*p*‐value	0.457
hs‐CRP (mg/L)	*R*	−0.425
	*p*‐value	<0.001
FBG (mg/dl)	*R*	−0.233
	*p*‐value	0.058
2hpp (mg/dl)	*R*	−0.205
	*p*‐value	0.097
Creatinine (mg/dl)	*R*	−0.068
	*p*‐value	0.584
LDL‐C (mg/dl)	*R*	−0.315
	*p*‐value	0.009
TC (mg/dl)	*R*	−0.082
	*p*‐value	0.508
TG (mg/dl)	*R*	−0.122
	*p*‐value	0.326
HDL‐C (mg/dl)	*R*	0.078
	*p*‐value	0.461
TG/HDL‐C ratio	*R*	−0.154
	*p*‐value	0.146
FMD (mg/dl)	*R*	0.154
	*p*‐value	0.212
HbA1c (%)	*R*	−0.012
	*p*‐value	0.923
A/C	*R*	0.226
	*p*‐value	0.066
BMI (kg/m^2^)	*R*	0.066
	*p*‐value	0.596
Fat (%)	*R*	0.024
	*p*‐value	0.847
Duration of DM (years)	*R*	0.066
	*p*‐value	0.596

Abbreviations: 2hPP: Two‐hours post‐prandial blood glucose; A/C: albumin/creatinine; BMI: body mass index; FBG: fasting blood glucose; HbA1c: glycated haemoglobin; HDL‐C: high‐density lipoprotein cholesterol; hs‐CRP: high‐sensitivity C‐reactive protein; LDL‐C: low‐density lipoprotein cholesterol; TC: total cholesterol; TG: triglycerides

According to simple linear regression analysis, FBG, LDL‐C, TC, TG and HbA1c were statistically significant predictors of FMD concentration (each variable separately).

We conducted multiple regression analyses to predict FMD based on age, irisin, hs‐CRP, fasting blood glucose (FBG), 2hpp, creatinine, LDL‐C, TC, TG, HbA1c, BMI and fat percentage. FMD is statistically significantly predicted by HBA1c, BMI and fat percentage only.

Stepwise regression was used to predict FMD (Table 4) based on age, irisin, hs‐CRP, FBG, 2 h postprandial, creatinine, LDL‐C, TC, TG, HbA1c, BMI and BIA. FMD was statistically predicted only by HBA1c, BMI and LDL‐C variables (*p*‐value <.05).

**TABLE 4:** Multiple stepwise regression analysis with FMD level as dependent variables

Multiple stepwise regression analysis with FMD level as dependent variables					
Models of stepwise regression		B	*p*‐value	95.0% Confidence Interval for B	
				Lower Bound	Upper Bound
1	(Constant)	39.470	<.001*	28.433	50.507
	HbA1c (%)	−2.451	<.001*	−3.639	−1.263
2	(Constant)	51.633	<.001*	37.095	66.171
	HbA1c (%)	−2.222	<.001*	−3.382	−1.062
	LDL‐C (mg/dl)	−.138	.017*	−.251	−.026
3	(Constant)	40.381	<.001*	22.946	57.816
	HgA1c (%)	−2.435	<.001*	−3.579	−1.292
	LDL‐C (mg/dl)	−.151	.008*	−.261	−.041
	BMI (kg/m^2^)	.571	.031*	.052	1.090

Multiple stepwise regression analysis with irisin level as dependent variables					
Models of stepwise regression		B	*p*‐value	95.0% Confidence Interval for B	
				Lower Bound	Upper Bound
4	(Constant)	6.516	<.001*	4.806	8.226
	hs‐CRP (mg/L)	−0.099	<.001*	−.151	−.047
5	(Constant)	9.523	<.001*	6.693	12.354
	hs‐CRP (mg/L)	−0.093	<.001*	−.144	−.043
	LDL‐C (mg/dl)	−0.031	.011*	−.054	−.007

*Note:* Bold denotes significant values.

Abbreviations: BMI: body mass index; HbA1c: glycated haemoglobin; hs‐CRP: high‐sensitivity C‐reactive protein; LDL‐C: low‐density lipoprotein cholesterol.

The level of irisin was predicted using multiple regression using age, FMD, hs‐CRP, FBG, 2hpp, creatinine, LDL‐C, TC, TG, HBA1c, A/C, duration of disease, BMI and BIA. This study found that only hs‐CRP, LDL‐C and A/C variables significantly predicted irisin levels (*p*‐value <.05).

According to a stepwise regression analysis of all other parameters (Table 4), only hs‐CRP and LDL‐C were statistically significant in their ability to predict irisin levels.

Based on BMI, study subjects were divided into three categories: nonobese individuals with a BMI less than 25, overweight individuals with a BMI of 25–29, and obese individuals with a BMI greater than 30. There were statistically significant differences between different groups regarding FBG, 2 h postprandial, HbA1c, LDL‐C, fat percentage and FMD variables, as illustrated in (Table 5).

**TABLE 5:** A comparison of laboratory and subject criteria based on BMI

	Normal (*n* = 53)	Over weight (*n* = 23)	Obese (*n* = 16)	*p*‐value
	Mean ± SD	Mean ± SD	Mean ± SD	
Irisin (ng/ml)	3.2 ± 1.5	3.6 ± 2.1	3.5 ± 1.9	.6
hs‐CRP (mg/L)	29.1 ± 8.1	31.9 ± 6.6	31.2 ± 9.7	.3
FBG (mg/dl)	144.4 ± 60.5	166.3 ± 41.9	195.7 ± 53.8	.005*
2‐hpp (mg/dl)	206.7 ± 88.5	233.8 ± 65.1	272.3 ± 58.4	.01*
Creatinine (mg/dl)	0.81 ± 0.14	0.84 ± 0.18	0.91 ± 0.18	.1
LDL‐C (mg/dl)	92.6 ± 19	100.9 ± 15.5	105.3 ± 20.7	.03*
TC (mg/dl)	186.7 ± 55.1	199.4 ± 45.4	221.5 ± 45.8	.06
TG (mg/dl)	128.9 ± 43.7	136.2 ± 49.1	186.6 ± 92.5	.05
HDL‐C (mg/dl)	74.9 ± 30	73.8 ± 36.4	84.9 ± 34.1	.5
TG/HDL ratio	2.07 ± 1.5	2.23 ± 1.7	2.23 ± 1.2	.9
FMD (mg/dl)	22.5 ± 10.7	17.6 ± 7.9	16.1 ± 9.1	.03*
HbA1c (%)	7.1 ± 2.2	8.9 ± 2.04	9.9 ± 1.6	.001*
A/C	19.8 ± 6.7	21.3 ± 7.02	23.1 ± 5.9	.2
Fat (%)	30.2 ± 6.4	33.8 ± 3.5	37.5 ± 4.1	.001*
Disease Duration (years)	5.6 ± 1.4	5.9 ± 1.9	5.1 ± 2.2	.4

*Note:* Bold denotes significant values.

Abbreviations: 2hPP: Two‐hours post‐prandial blood glucose; A/C: albumin/creatinine; FBG: fasting blood glucose; HbA1c: glycated haemoglobin; HDL‐C: high‐density lipoprotein cholesterol; hs‐CRP: high‐sensitivity C‐reactive protein; LDL‐C: low‐density lipoprotein cholesterol; TC: total cholesterol; TG: triglycerides.

The men and women in our study were classified according to their body fat percentage by BIA into the following categories: very low body fat, low body fat, optimal body fat, moderately high body fat and high body fat. As assessed by BIA, we noted a non‐statistically significant difference in body fat percentage between males and females. Most study participants fell into the moderate‐ to high‐fat percentage category. Despite higher irisin levels in patients with high body fat percentages, these differences were not statistically significant (*p*‐value .819 and .235 in males and females, respectively). This study did not include insulin resistance, as it fell outside the scope of its purpose, which was to evaluate serum irisin levels in relation to ED among people with T2DM.

## 4 DISCUSSION

Several metabolic biomarkers have been investigated as possible regulators of glucose homeostasis in response to the growing prevalence of metabolic diseases associated with obesity, including T2DM.^25^ Diabetes and insulin resistance are correlated with irisin levels, indicating that macroangiopathy may also be associated with low irisin levels in patients with T2DM.^26^

Most laboratory and patient criteria were statistically different between patients with T2DM and controls (*p*‐values <.05). This study found no statistically significant difference in circulating Irisin levels between patients with T2DM and healthy controls, in contrast to previous studies which showed lower levels of Irisin in patients with a new diagnosis of T2DM.^24^

According to ***Choi* et al.**, adults with newly diagnosed T2DM have lower levels of irisin than those with normal glucose tolerance. This shows statistically significant inverse associations between irisin concentrations and the development of T2DM.^21^ Furthermore, Liu et al. found that, regardless of age, gender and body mass index, irisin levels were significantly decreased in adults with T2DM.^27^ A number of other studies have demonstrated contradictory results. This suggests that irisin levels are affected by a variety of body factors, including glucose and fatty acids, in patients with T2DM. According to Kurdiova et al., in vivo and in vitro studies were conducted, with opposite effects found; in the in vivo study, irisin and mRNA expressions of FNDC5 in skeletal muscle and adipose tissues were reduced, but FNDC5 expression in myotubes from the in vitro study was significantly higher.^28^

Based on our results, we found a statistically significant negative linear relationship between irisin and hs‐CRP as well as LDL‐C. Hs‐CRP is a marker of inflammation for CVD, which is consistent with Park, et al.'s study which found that patients with low irisin levels and hyperglycaemia were at an increased risk of metabolic syndrome and CVD. In addition, irisin was found to have a protective effect against coronary artery disease.^29^

Our results regarding ED demonstrated a statistically significant negative correlation between FMD and A1c, FBG, LDL‐C, TG, TG/HDL‐C ratio, TC, indicating that high blood glucose and dyslipidaemia were highly predictive of positive results with FMD and ED. Similarly, Yan et al. found that serum irisin was significantly negatively correlated with HbA1c in obese Chinese adults.^30^

In our study, there was a negative linear correlation between FMD and LDL‐C which means that increasing LDL‐C level means more dyslipidaemia and, decreasing FMD and also increasing ED. Also, there was a negative correlation between FMD and cholesterol levels as when increasing hyperlipidaemia decreasing FMD accordingly. These findings were in line with the reported LDL‐C measurements in the prediction of changes in cardiovascular disease in patients with diabetes and evaluation of intima‐media thickness (IMT) that occurred in diabetic patients as assessed by FMD with LDL.^31^

Regarding the relation between different FMD levels with Irisin, we found that most of the patients who had severe FMD (less than 10) had reduced irisin levels, while other patients with mild or moderate FMD had different ranges of irisin levels, some had reduced irisin level, and others had normal irisin level. Following our results, the reported circulating irisin levels were positively correlated with endothelium dysfunction, and significantly reduced in newly diagnosed T2DM without clinical angiopathy, and also, irisin levels could be a marker for detecting early‐stage of angiopathy in T2DM.^24^

In the current study, there was a negative linear correlation between FMD and HbA1c and with FBG, which means that poor glycaemic control is linked to more ED and complicated by atherosclerosis so accordingly decreasing FMD. It had been reported that ED has a positive association between circulating irisin levels, fasting and 2hpp.^32^, ^33^, ^34^ It has been reported that impaired insulin clearance is a major determinant of hyperinsulinemia,^35^ and therefore, it is led to an increase in fasting and 2hpp blood glucose.

Serum Irisin level was affected by increasing FBG, which can be agreed with ***Liu* et al.*,*** who observed an inverse relationship between hyperglycaemia and circulating irisin levels.^27^ Also, ***Park* et al.*,*** reported that circulating irisin levels were negatively correlated with FBG levels.^29^ On the other hand, ***Stengal* et al.** did not find a correlation between serum irisin and blood glucose levels but found that serum irisin was positively correlated with insulin levels.^36^

By comparing the serum levels of irisin between T2DM patients with and without MVD (macrovascular diseases), it was found that irisin decreased more significantly when MVD existed. The findings still suggested that irisin would be a potential target for monitoring and intervention of T2DM and its associated vascular complications.^37^

Irisin is positively correlated with triglycerides, TC and LDL‐C. We studied T2DM patients according to BMI and percentage of body fat using bioelectrical impendence analysis and noticed that serum Irisin level increased in obese patients, which was explained by other studies as increasing adipose tissue (which releases Irisin beside skeletal muscles) in increased irisin level which matched with other studies. Similarly, Liu et al. found a positive correlation between BMI or body fat mass and circulating irisin levels.^27^ Also, *Park* et al. reported that circulating irisin levels were positively associated with body fat mass in the whole cohort and with BMI in the subgroup of obese individuals.^29^

Irisin levels were correlated with body mass index (BMI), and fat mass and authors described adipose tissue as the main source of circulating irisin in such patients.^38^ Other authors have also found a positive correlation between BMI and fat mass.

In obese individuals with other cardiovascular risk factors, higher serum irisin levels were found to be correlated with high BMI.^32^ Irisin has also been found to be positively correlated with triglycerides and total cholesterol.^39^

The results showed higher body fat, visceral fat and lower muscle mass in diabetic patients and that circulating irisin was positively associated with muscle mass. Recently, some studies showed that irisin might be associated with the protection of endothelial function.^24^

We reported that the obesity prevalence is generally higher in women than in men in all age groups, and there is also a sex difference in body fat distribution. Sex differences in obesity can be explained in part by the influence of gonadal steroids on body composition and appetite; however, behavioural, socio‐cultural and chromosomal factors may also play a role.^38^

Several studies have investigated the relationship between circulating irisin and BMI. In a study of 117 healthy middle‐aged women with BMI ranging from 20.0 to 47.7 kg/m^2^, circulating irisin had positive associations with fat‐free mass and a positive trend with BMI.^32^ Obese patients have higher circulating irisin levels compared with normal‐weight controls and anorexic patients, and irisin has a positive correlation with body weight and BMI. The current study has some limitations. The sample size was relatively small, with a relatively limited age range of participants. The study design was a case–control design that did not allow for analysing the response of serum irisin to exercise or training since irisin is a myokine that is induced by exercise.

## 5 CONCLUSIONS

In conclusion, the results of the study suggest that serum irisin may have clinical utility as a predictive measure for early cardiovascular disease. Therefore, it may be used as a new early indication of ED that can prevent cardiovascular disease from progressing. As irisin is a relatively new marker of ED, it may be worthwhile to conduct additional studies on irisin that take into account endothelin, prostaglandins, angiotensin II, E selectin and adhesion molecules in the future. A future research study should explore the role of irisin in insulin resistance, atherosclerosis and diabetes complications.

## AUTHOR CONTRIBUTIONS

**Thoraya Mohamed Ahmed:** Investigation (equal). **Mahmoud Nassar:** Writing – original draft (equal). **Hanaa Ahmed Abdallah Mohamed:** Investigation (equal). **Khaled E ElHadidy:** Supervision (equal). **Hanan Mohamed Farhan:** Investigation (equal). **Ahmed Sayed Abd El Basset:** Investigation (equal). **Riem M Elmessiery:** Writing – original draft (equal). **Mahmoud Farid Kamel:** Methodology (equal).

## FUNDING INFORMATION

No funding for this research.

## CONFLICT OF INTEREST

No conflict of interest.

## ETHICAL APPROVAL

The manuscript has been read and approved by all the authors, the requirements for authorship, as stated earlier in this document, have been met and each author believes that the manuscript represents honest work.

## Data Availability

The data that support the findings of this study are available from the corresponding author upon reasonable request.
